# One Year on: The Impact of COVID-19 on the Lives of Freelance Orchestral Musicians in the United Kingdom

**DOI:** 10.3389/fpsyg.2022.885606

**Published:** 2022-05-30

**Authors:** Susanna Cohen, Jane Ginsborg

**Affiliations:** ^1^Interdisciplinary Department of Social Sciences, Bar Ilan University, Ramat Gan, Israel; ^2^Centre for Music Performance Research, Royal Northern College of Music, Manchester, United Kingdom

**Keywords:** coronavirus, portfolio career, identity, music performance, post-traumatic growth, self-employed

## Abstract

Before the drastic disruption caused by the sudden emergence of the COVID-19 pandemic, 85% of the United Kingdom’s 14,000 orchestral musicians were self-employed freelance workers, engaged in busy and varied portfolio careers comprising a combination of orchestral, West End theatre, chamber music, and commercial recording work. Between May and June 2020 we carried out a first study examining the impact of the pandemic on the lives of 24 self-employed orchestral musicians, all established freelancers. Twelve were mid-career and 12 were late-career (described in that study as “seasoned”). They all reported having lost their much-loved performing careers, missing music making and colleagues, and being anxious about the future of the music profession. However, there were some differences between the two groups: the late-career participants demonstrated greater financial and emotional resilience, while the mid-career musicians reported distress, confusion, and anxiety about their identity as musicians. In the present follow-up study, we aimed to examine the impact of the first year of the pandemic on the lives of 21 of the same musicians. We found that while all the mid-career participants remained committed to their performing careers, many late-career participants aged 54–59 had developed interests in non-performing music work, and the older late-career participants, aged 65 and over, feared that they might already, *de facto*, have retired. We discuss the findings with reference to the precarity of freelance orchestral musicians’ lives, lifespan models of musicians’ careers, self-determination theory and post-traumatic growth, and their implications for music colleges and musicians’ support organizations.

## Introduction

The COVID-19 pandemic has drastically disrupted the performing and creative arts industries in the United Kingdom (Creative Radar, 2021; Spiro et al., 2021). Whereas the internationally respected UK music industry contributed £5.8 bn to the UK economy in 2019 before the pandemic, this sum fell by 46% to £3.1 bn in 2021, and the number of people supporting themselves by working full time in music fell by 35%, from 197,000 to 128,000 (UK Music, 2021). Pre-pandemic, there were approximately 14,000 professional orchestral musicians in the United Kingdom (Association of British Orchestras, (ABO), 2019). Unlike most European countries where they tend to be salaried, over 85% of orchestral players in the United Kingdom were self-employed as members of freelance orchestras, on short term contracts as extra or deputy players, or on an *ad hoc* basis (Teague and Smith, 2015; Association of British Orchestras, (ABO), 2019; Willis et al., 2019). These freelance musicians built busy portfolio careers comprising a rich and varied combination of performing activities including orchestral and chamber music, West End shows, community work, commercial and recording sessions, and teaching (Bartleet et al., 2019). Although some studies carried out before the pandemic found that self-employment is more strongly associated with job and life satisfaction than employment by a single employer (Warr, 2018), other studies have shown that, for freelance classical musicians, lack of financial security associated with self-employment is linked to increased anxiety (Dobson, 2010; Oakland et al., 2012; Petriglieri et al., 2019).

Classical musicians tend to be highly motivated (McPherson and Zimmerman, 2011; McPherson et al., 2019), having spent thousands of hours developing their skills (Ericsson et al., 1993), and are deeply emotionally invested in and identified with their work (Teague and Smith, 2015; Bonneville-Roussy and Vallerand, 2020). Pre-pandemic, freelance orchestral musicians enjoyed high levels of wellbeing (Ascenso et al., 2017) and job satisfaction (Brodsky, 2006; Gembris et al., 2018). Given, however, that self-employed freelance musicians often experience anxiety about the insecurity of their jobs (Bennett and Hennekam, 2018) and that musicians, generally, can find involuntary career transitions traumatic and a threat to their wellbeing (Maitlis, 2009; Oakland et al., 2012; Hennekam and Bennett, 2016), freelance orchestral musicians were likely to be very vulnerable when faced with the sudden loss of work and career brought about by the pandemic.

Lifespan models of orchestral musicians’ working lives show that they are often very long (Allmendinger et al., 1996; Brodsky, 2011) and can be divided into three different stages (Manturzewska, 1990; Bennett and Hennekam, 2018; López-Íñiguez and Bennett, 2020). The first stage is early career (approximately ages 20–30, with fewer than 10 years of professional experience), characterized by the challenge of establishing oneself in the profession and the search for employment. The second is mid-career (approximately ages 30–45, with 10–25 years of professional experience), described as “a stage of artistic and professional expansion, and of the greatest achievements” (Manturzewska, 1990, p.135), and also characterized by the challenge of consolidating one’s professional achievements and the difficulty of obtaining a work/family balance (Teague and Smith, 2015). The third is late career (approximately age 55 and over, with 25 or more years of professional experience), when musicians concentrate more on teaching, social responsibility, acknowledging their achievements, and potentially managing a decline in their ability to perform (Gembris et al., 2018). Manturzewska (1990) suggests that musicians tend to “surge forward in their musical career until they are in their mid-forties and then begin to register the first signs of physical and psychic fatigue” (p. 136), at which point a shift begins, from a focus, mid-career, on their own performing achievements to an increased interest in teaching and a greater sense of social responsibility (Manturzewska, 1990; Bennett and Hennekam, 2018), which peaks between the ages of 55 and 65 in a period of optimum teaching achievement.

To investigate the impact of the first lockdown and its immediate aftermath on the lives of freelance orchestral musicians, we conducted an interview study in May and June 2020 of 12 mid-career (aged 35–45) and 12 late-career freelance orchestral musicians (aged 53 and over, but with more than 25 years’ professional experience. In that study, we referred to these participants as “seasoned”). We were interested in examining whether previously successful mid-career musicians interrupted in the middle of what would normally be a period of professional expansion and peak performance might be experiencing this challenging period differently from those who had already enjoyed many years of successful professional achievements. The aims of that first interview study were to examine (1) how freelance orchestral musicians were experiencing the impact of the pandemic on their lives, and (2) the similarities and differences between the experiences of mid- and late-career musicians. As we wanted to investigate the impact of the pandemic on musicians who had already established successful, busy, and freelance careers prior to the sudden arrival of the pandemic, we didn’t include in our sample early-career musicians, who might still have been in the process of trying to establish their careers as freelancers.

In that first study (Cohen and Ginsborg, 2021), we found that the first lockdown had brought about enormous, negative changes and challenges to the lives of the participants; without exception, they felt the sudden loss of their much-loved, previously successful careers, and anxiety about their finances and future careers. While these findings reflected those of a large survey of people working in the performing arts in the United Kingdom (Spiro et al., 2021), we also found some differences between the mid- and late-career groups: mid-career participants reported greater emotional distress, more anxiety about money, less engagement in music making, and more confusion about their identity as musicians and their future careers than late-career participants, creating an overall picture of a group of mid-career musicians in crisis. By comparison, late-career participants felt they had already achieved a great deal. They were less anxious about money, and many were more focused on teaching and helping their students. As a group, they demonstrated greater emotional and financial resilience.

More than two years on, the pandemic is still with us. Subsequent lockdowns, social distancing rules and restrictions on live performance continued throughout much of 2020 and 2021 in response to new variants of the SARS-Cov-2 virus and ever-changing infection rates [World Health Organization (WHO), 2021].^1^ A year after we conducted our study there was still very little work for musicians giving live performances (BBC, 2021a), and as a consequence, freelance musicians’ lives and livelihoods were still very far from returning to normal. The impact of the pandemic has been found to be more severe on people who are self-employed than employees. Incomes and profits from self-employment are well below pre-pandemic levels (Blackburn et al., 2021), and people who are self-employed now experience lower happiness and wellbeing than their counterparts in employment (Yue and Cowling, 2021). We therefore conducted a second, follow-up interview study to examine the longer-term impact of the pandemic on the lives of the participants in the first study. Its aims were to examine (1) how these freelance orchestral musicians had experienced and were still experiencing the pandemic in the year since they were first interviewed and (2) the similarities and differences between the experiences of the two groups of mid- and late-career musicians.

## Materials and Methods

### Participants

Of the 24 participants in our first study, all but one responded to the invitation to participate in the present study. All 12 of the mid-career group and nine of the late-career group took part in a single one-to-one interview on Zoom. One of the remaining three was unable to participate due to health issues, the second was no longer performing but working as a full-time teacher and administrator in a music college, and the third didn’t respond. In our first study, mid-career participants had to be aged 35–45 in 2020, with a minimum of 10 years of professional playing experience; a year later they were aged 36 to 46 (*M* = 41.75, SD = 3.96) with an average of 18.92 years of professional playing experience (SD = 3.68). Similarly, late-career participants had to be aged 50 or older with a minimum of 25 years of professional playing experience; a year later, they were aged 54 and over (*M* = 61.78, SD = 7.34), with an average of 38.22 years (SD = 7.22) of professional playing experience. In the present study, four of the late-career group were aged 66 or more; we refer to these individuals as older late-career participants. The overall age range was 36 to 74 (*M* = 50.33, SD = 11.54), with an average of 27.19 years (SD = 11.14) of playing experience. There were 12 women and 9 men, 13 string, 5 woodwind, and 3 brass players (see Table 1 for participants’ demographic details). They all met the remaining inclusion criteria for our first study, designed to ensure that participants’ careers, pre-pandemic, were relatively homogenous: they were registered as self-employed, based within an hour’s drive of London, and had earned at least two-thirds of their income from music performance.

**Table 1:** Summary of participant information.

Career stage	Participant	Instrument family	Age range (years)	Professional playing (years)	Children at home/ dependants
Mid-	M1	Brass	35–40	16	–
career	M2	Strings	35–40	17	–
	M3	Woodwind	41–45	22	1
	M4	Brass	41–45	18	2
	M5	Strings	41–45	21	2
	M6	Woodwind	46–50	23	–
	M7	Brass	46–50	24	4
	M8	Strings	41–45	22	–
	M9	Woodwind	35–40	13	–
	M10	Strings	41–45	19	2
	M11	Strings	35–40	13	–
	M12	Strings	46–50	19	1
Late	L2	Strings	66+	41	–
career	L3	Strings	66+	34	2
	L4	Strings	56–60	34	2
	L5	Woodwind	66+	51	–
	L6	Strings	51–55	33	–
	L8	Woodwind	66+	49	–
	L9	Strings	51–55	31	3
	L11	Strings	56–60	35	1
	L12	Strings	56–60	36	–

M, Mid-career musician and L, late-career musician.

### Procedure and Data Collection

Ethical approval having been granted by the RNCM Research Ethics Committee, we invited the 24 participants in the first study *via* email and/or Facebook Messenger to take part in single, one-to-one follow-up interviews with the first author on Zoom. These took place between June and August 2021, lasted approximately one hour, and were recorded on Zoom. Each video recording was deleted immediately after the interview and the audio recording was saved on the first author’s password-locked computer. The interview was semi-structured and contained questions focusing on the impact of the pandemic on participants’ lives over the past year and their thoughts about the future. Participants were asked if they were currently performing and about their experiences of doing so; how they were managing financially; and about their hopes and fears about continuing to work in the music profession. The full interview guide can be found in Appendix A.

### Data Analysis

The interviews were transcribed verbatim and analyzed using a five-stage inductive, recursive thematic approach (Braun and Clarke, 2006, 2019). The first author took the lead; the second author analyzed five (just under 25%) of the interviews independently. First, they read through the transcripts several times. Second, they coded the transcripts for meaningful phrases within and between the participants. Third, they grouped the codes into themes characterizing the impact of the pandemic on musicians’ lives. Fourth, they grouped the themes into overarching themes and examined similarities and differences between themes arising from the interviews with the two groups of participants. Fifth, they discussed, reviewed, and agreed on the final codes, themes, dominant theme, and overarching themes, and the similarities and differences between the mid- and late-career participants. The criterion of trustworthiness (Lincoln and Guba, 1986) was met by involving both researchers in the analysis; this also allowed for a richer and more nuanced reading of the data (Braun and Clarke, 2019) than if only one researcher had carried out the analysis.

## Results

**Figure 1:**
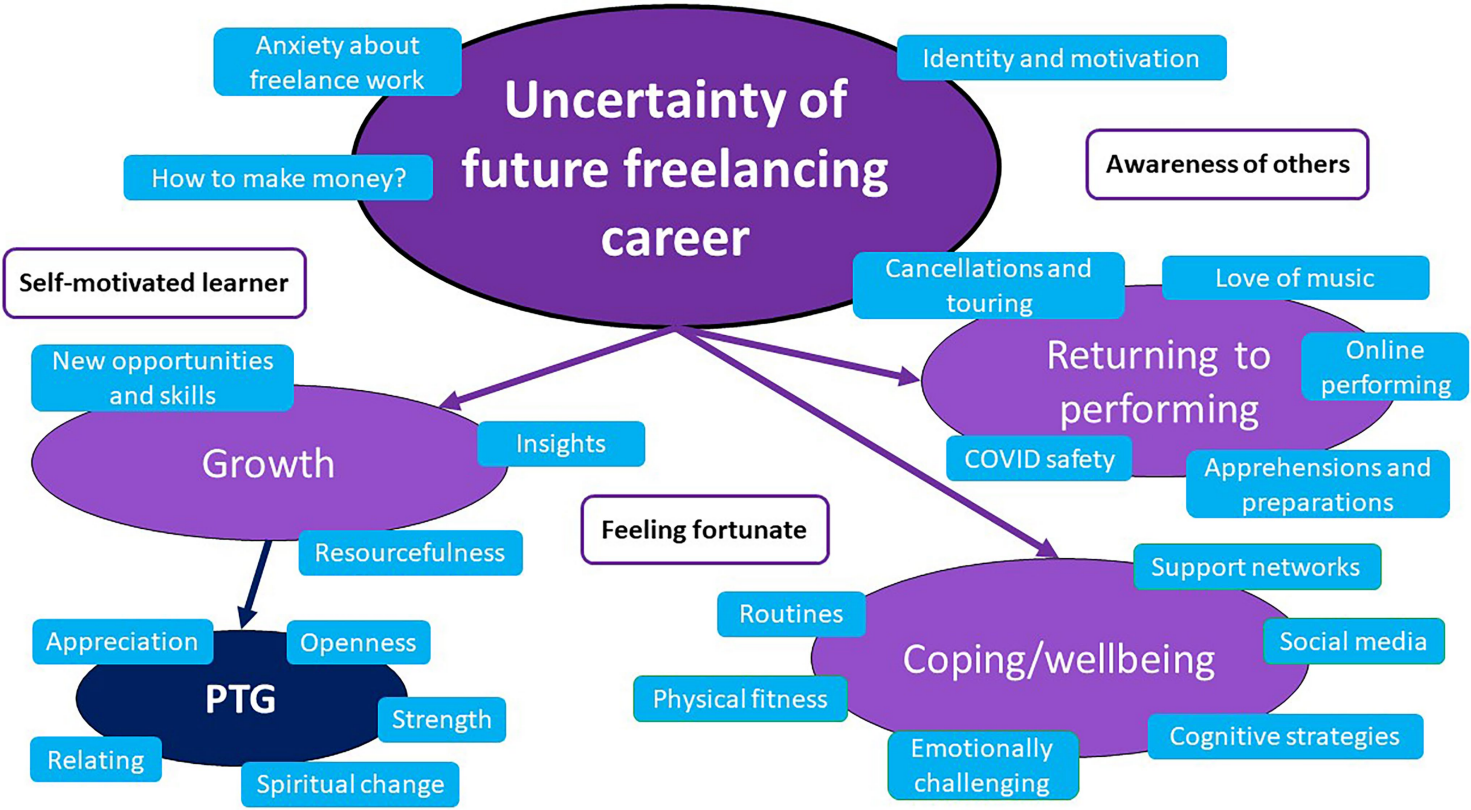
Model of themes and sub-themes.

The analysis produced a final set of 22 sub-themes that clustered into one central dominant theme *Uncertainty of future freelancing career* and three lower-order overarching themes: (1) *Returning to performing*, (2) *Coping strategies and maintaining wellbeing,* and (3) *Growth.* Three further latent themes were identified: *Awareness of others, Self-motivated learner,* and *Feeling fortunate.* These themes are summarized in Table 2 and represented graphically in Figure 1, illustrating the centrality of the dominant theme *Uncertainty of future freelancing career*. Quotations from interviews in the description of each theme, below, are attributed to mid-career (*M*) and late-career participants (*L*), respectively.

**Table 2:** Central dominant theme, overarching themes, sub-themes, group, and number of participants for each sub-theme.

Overarching themes	Sub-themes	*N* (Total)	*M*	*L*
Central dominant theme: Uncertainty of future freelancing career				
	Anxiety about fragility of freelance work	21	12	9
	Cancelations and touring	20	12	8
	How to make money?	18	12	6
	Maintaining identity and motivation	21	12	9
Overarching theme 1: Returning to performing				
	Love of music and colleagues	21	12	9
	Apprehensions and preparations	15	9	6
	COVID safety	21	12	9
	Online performing	20	12	8
Overarching theme 2: Maintaining wellbeing and coping strategies				
	Emotionally challenging	18	11	5
	Support networks	16	9	7
	Cognitive strategies	18	11	7
	Routines	12	6	6
	Exercise and physical health	14	7	7
	Social media	10	3	7
Overarching theme 3: Growth				
	New opportunities and skills	15	10	5
	Resourcefulness	15	11	4
	Insights	18	10	8
Early signs of post-traumatic growth (PTG)				
	Appreciation of life	8	2	6
	Openness to new possibilities	13	9	4
	Personal strength	8	6	2
	Relating to others	9	6	3
	Spiritual change	4	2	2
Latent themes				
	Awareness and concern for others	20	11	9
	Being a self-motivated learner	21	12	9
	Feeling fortunate	21	12	9

N, total number of participants mentioning theme, M, number of mid-career participants, and L, number of late-career participants.

### Uncertainty of Future Freelancing Career

This dominant theme was linked to the three lower-order overarching themes and encompassed four groups of sub-themes: *Anxiety about the fragility of freelance work, Cancelations and touring, How to make money,* and *Maintaining identity and motivation.*

All 21 participants described *anxiety about the fragility of freelance work,* and all but two had done almost no performing in the year since their first interview: “I had one date, yeah. You know, you are talking nothing really” (M11). Many were very anxious about whether and when freelance work would resume, and one was finding the current situation intolerable: “I feel that I can’t, I can’t carry on doing what I do as a freelancer, because I, this year has been absolutely devastating […] I love playing, I really do, and I can’t imagine not playing [my instrument], but I also can’t imagine being this stressed and this unhappy for the rest of my life” (M1). At the time of the follow-up interviews, lockdown and social distancing restrictions had started to ease according to the delayed Step 4 of the UK government’s roadmap (Institute for Government, 2021) and, while participants reported now having a small number of tentative bookings, they would be insufficient to sustain a career. Participants observed that there were fewer freelance work opportunities than pre-pandemic, partly because the self-employed members of major orchestras were currently doing all the work offered to them thus leaving little deputizing work for freelancers, and partly because symphony orchestras were unable to book extra string players to boost their string sections (a regular practice pre-pandemic) because of the space restrictions imposed by compliance with social distancing rules: “I used to do a lot of freelance extra playing with the major sort of symphony orchestras and that is not happening, because they can’t get enough strings on the stage to use their own sections, let alone to use extras” (M5).

Several participants reported that conditions and fees had deteriorated as competition for limited opportunities increased. One string player worked on a West End show: “In these times, beggars can’t be choosers. So, obviously I show up, smile my best smile and play as well as I can […] What is a shame is that behind every freelance musician is another even more desperate musician, willing to step up at any moment, to work for practically nothing” (M12). Another was offered “exposure” in lieu of a fee for performing but turned it down: “We definitely expect to be paid. If the catering staff are being paid, we expect to be treated fairly, and not [for management] to take the mick, just because we’ve lost a lot of work” (M2).

Many of the participants were damning about the government’s lack of support for the performing arts and understanding of their work: “At no point in any of this […] has anything that I do been (a) recognized, or (b) valued or (c) understood” (L11); “We are the ones who’ve been most broadly shat on […] classical music doesn’t get mentioned really. Do we exist? Oh god!” (L6). They despaired at the general public’s lack of awareness of what it means to work in the music industry, despite evidence that 74% of more than 1,000 adults surveyed for UK Music reported that music listening is important to their quality of life, 57% said that it had helped them cope during lockdown, and 39% said that music had become more important to them over the past year (Public First, 2021). “The biggest, almost kick in the teeth really, is that people do not know where it [music] comes from […] I’ve had somebody say to me ‘oh why don’t you just get another job?’ I said ‘well, I have trained for an awfully long time to do this, and also if the music profession stops, where’s all the music on the TV, the films, your gym list where is all the music going to come from?’” (M8).

All participants reported *cancelations* of work at short notice, with no cancelation fee. This was particularly distressing, given the scarcity of work: “Those are the only concerts I’ve got […] I’ve got nothing else, it seems, it feels like a much bigger deal if it is cancelled. Partly because of the money, but also just the wanting to play and wanting to perform” (M8). They acknowledged that the circumstances were exceptional and that orchestral fixers had to use “force majeure” clauses when making last-minute alterations due to illness, quarantine, and ever-changing rules and timelines (Institute for Government, 2021), but many complained that there was no reciprocity, and orchestral managers and fixers lacked sensitivity when canceling work. One participant experienced repeated cancelations although she had been a loyal Principal player with the same orchestra for many years:

If I don’t say “yes” to at least 70% of the work they offer me, I’m out of the orchestra […] I feel so bound to have to take the work they offer me, but at the same time, they are clearly not bound to keep the dates they give me […] I’ve put in all the work to be prepared, and then the orchestra have just turned round and said “oh sorry, we’re changing the programme”[…] it’s just devastating not being part of it […] I rang management and cried on the phone […] and said “this can’t be, you’ve cancelled all of our work […] what are we meant to do, how are we meant to survive? (M1).

Several participants described double- or even triple-booking themselves, in the hope that at least one piece of work might materialize: “Usually I would have just gone ‘well I’ll just take the one that I really want to do’ […] but I just thought, ‘well actually, if you’re going to put me on hold, I’m going to put you on hold as well’ […] so I took everything on, and waited to see what the restrictions would do” (M3). In the current climate, musicians are expected to be flexible and to understand that dates may have to be postponed or canceled without compensation. It would be helpful, during this period, if orchestral managers and fixers could reciprocate by not penalizing musicians whose availability may change at short notice.

They all expressed concerns about *touring*, with only four of the mid-career group (three women and one man) having performed outside the United Kingdom since the start of the pandemic: “I think the combination of Brexit and COVID has absolutely knocked the whole of foreign touring on the head” (L8). Many commented that the pandemic was currently masking the impact of Brexit, and they were all concerned that once the pandemic was over, working abroad would be much harder because of Brexit: “If you were going to be asked for a festival, they’ll just see your name or your nationality and just think ‘oh it would just be so much easier to ask this French [player] or this German [player]’” (L9). The Musicians’ Union (MU) is now campaigning for the United Kingdom government to provide a special musicians’ passport in their #WorkingInTheEU campaign (Musicians’ Union, MU, 2022a), to ease the difficulty of working in the EU post-Brexit.

In the first study, which took place during the first lockdown, nearly all the participants were extremely concerned about *how to make money*: “I went into complete like survival mode when the whole thing happened, and I was like ‘What are we going to do? How are we going to eat? How are we going to pay the bills?’ We had to do the universal credit^2^ at the beginning as well […] I felt awful” (M3). In the present study, only one mid- and one late-career participant said that their earnings from performance had returned to their pre-pandemic levels. They had all managed financially, however, typically from a combination of government grants, state pensions, teaching, non-music work, using savings, and cutting back on spending.

Twenty-one of the original 24 participants had applied for government self-employment income support scheme (SEISS) grants.^3^ Of these 21, eight (four men, four women) were ineligible and therefore struggled financially. This figure reflects the 38% of respondents to the MU’s much larger survey who were in the same position and suggests that the criteria for eligibility should now be revised. Recipients of SEISS grants were grateful, although several had found it complicated to apply for the fifth one, and two were worried that applying for an SEISS grant might affect future applications for a mortgage. Four of the older late-career participants (three men and one woman) received a state pension, but nearly all the late-career group considered themselves better off, financially, than their younger colleagues: “I think it’s less of an issue for somebody like me than for somebody younger, who I mean, yeah, I’m very well heeled, with a house and instruments and stuff like that” (L6).

Teaching provided a major source of income for most of the participants (*M* = 11, *L* = 6). Nine of the mid-career group (seven women and two men), but none of the late-career group, had taken on extra teaching to make up for lost income from performing. Most valued teaching not only for the regular income it provided but also for the opportunity to carry on working as a musician: “I’m hoping that the bits of teaching work […] is sort of going to you know, keep the wolf from the door because it’s what I’ve tried to have as security” (M3). However, four mid-career female participants were unhappy about teaching full time in place of performing: “I’m very fortunate in that I’m working pretty much full time, but it’s all teaching, which is not what I want to be doing, but it has kept a roof over our heads […] I don’t really like it that much […] so that’s quite tough to sort of look at” (M5). By contrast, five late-career participants (three women and two men) reported providing their music college students with extra lessons, in addition to much-needed emotional support, because, in some cases, they were the only people their students (particularly those from overseas) were seeing, in person, from one week to the next. This demonstrates their commitment to teaching, supporting lifespan models suggesting that in the later stages of their careers musicians tend to focus on teaching and taking social responsibility (Manturzewska, 1990; López-Íñiguez and Bennett, 2020).

Nearly a third of the participants (*M* = 2, *L* = 4) had developed other income streams from non-performance but music-related activities, including writing articles for a music teachers’ magazine, using music-based activities when teaching English as a foreign language, museum curation, and selling instruments. Two were beginning to use music more therapeutically and two had taken advantage of the government bounce-back loan scheme: one to buy new recording equipment so as to perform online and the other to keep their instrument-selling business afloat. Three mid-career participants had worked in the hospitality industry (e.g., in pubs, bars, and cafés) to support themselves, and three had been couriers doing food deliveries: “I was in floods of tears by the end of my shift when I was handed the mop and bucket to mop the gents’ toilets floors of the pub, and I thought, I was doing it and I thought ‘what have I [done], I can’t believe 12 years [of professional playing] has come down to this” (M11). Other mid-career participants started a home distillery business, sold their landscape paintings, re-qualified as a teacher of English as a foreign language, and moved to a European country (see “Growth”).

The question of *maintaining identity and motivation* as a musician when unable to earn a living from performing arose in all the follow-up interviews. A year earlier it had been predominantly the mid-career participants who were in crisis about their professional identities, with seven actively considering leaving professional performing and the late-career participants appearing more emotionally and financially robust. Now, however, all 12 mid-career musicians were still committed to continuing careers as performers: “It doesn’t feel like it’s right for me to leave music, because this is, this is my lifeblood” (M12). The late-career participants were less committed. Besides the two who contributed to the first but not the present study, one had suffered serious injury and was not currently playing. Three older participants (one woman and two men), aged 66 and over, were contemplating retirement: “It’s been difficult to hang on to the idea that I am a professional musician, quite honestly […] I thought, ‘gosh I’m not sure I really want to go on doing this’” (L2); “I have reservations about struggling with work, having to make the profession work for me […] I don’t think I could go back to running around like I did before […] the idea of jumping in the car and driving to Truro^4^ to do one concert and then driving back seems like an effort […] I know that I don’t really want to retire yet, which is not to say that I might not retire” (L3) (see *Apprehensions and preparations*, below).

All the participants had continued to practice their instruments over the past year, although nearly half (*M* = 5, *L* = 5, five men and five women) described periods of struggling with motivation in the absence of performing opportunities: “I can’t say I enjoy it very much at the moment. I mean I do it because that’s what you do, it’s like you brush your teeth in the morning, it’s unthinkable just not to play” (L8); “This year, love for [my instrument] just went out the window, just the lack of work […] it was all very doom and gloom. It just got to the point where I thought ‘what’s the point? what am I practising for?’” (M11). One mid- and one late-career participant were enjoying having more time for playing and developing their skills: “I was getting study books out that I wouldn’t normally have had time for […] the motivation just was always there” (L9). Others wanted to be “match fit” for demonstrating to students and returning to performing when possible; indeed, several mid-career participants had organized online performances as a way of ensuring that they would have to practice, and one was a member of a support group in which musicians reported their daily practice to each other. In this way, they demonstrated both resourcefulness and understanding of their own motivations (López-Íñiguez et al., 2022).

### Returning to Performing

The overarching theme *Returning to performing* comprised four sub-themes: *love of music and colleagues*, *apprehensions and preparations*, *working in a COVID-19 safe environment,* and *online performing*.

Participants were emotional when they spoke about their *love of music and colleagues*. Of their few experiences of working during the past year, one said, “It was joyful and incredibly emotional, there were a lot of us bawling our eyes out all the way through (laughs), but it felt like a, just a snippet of our old lives, who we were, what we’re supposed to be doing, and I sort of feel a bit goose-bumpy even talking about it now. It’s, it was just, it was really wonderful, really wonderful” (M8). Such accounts illustrate musicians’ love for and emotional investment in their work (Bonneville-Roussy and Vallerand, 2020).

Participants’ *apprehensions and preparations* for returning to performing work centered on being able to play and dealing with the logistics of freelancing after such a long break. Many (*M* = 9, *L* = 5, seven men and seven women) doubted their competence: “I thought ‘I’m not sure I can still play this.’ I certainly used to be able to play it, I’ve done it lots of times, but erm, I, I practised it quite a lot, which, I don’t normally do […] I did find stamina a real issue” (L8). Others experienced music performance anxiety, known to be widespread among professional orchestral musicians (Kenny et al., 2014; Cohen and Bodner, 2019): “Walking back in there, I felt nervous, sick, sounds a bit strange […] but I felt nervous seeing colleagues again after such a long time […] playing in front of people” (M8). The mid-career participants were able to overcome their initial apprehensions, one saying “I was worried to begin with: ‘am I going to be able to play [my instrument]?’ but it quickly came back” (M3). This was not the case for three of the older late-career participants: “I am actually sort of wondering whether I really want, maybe the time has come, just to stop completely, you know. Suddenly the stresses and strains of concerts coming up after 15 months without anything, erm, it all looks a bit different” (L2); “I would feel very differently if I was 20 or 30 years younger, because I’d feel incredibly frustrated that I wanted to get out there playing, you know, have all this energy and stuff wanting to play. And I don’t feel that now at all, you know, I feel I’ve done it you know, and so it’s not like I’m itching to get back to it at all” (L8). These comments illustrate the change in focus that can occur in the late stage of a musician’s career (Manturzewska, 1990; López-Íñiguez and Bennett, 2020).

Pre-pandemic, retirement from orchestral life was already an issue of concern (Brodsky, 2011). In the absence of a statutory retirement age, both musicians and managements are uncertain when and how retirement should take place, and discussions between them can be difficult; there has been an increase in claims of unfair dismissal on the basis of age discrimination (e.g., Bonfiglio, 2018). Further research is urgently needed to identify the needs of both parties and develop guidelines addressing their expectations, setting out procedures, and ensuring appropriate support for musicians at this stage of their careers.

Logistics were a source of anxiety for five mid-career participants (three women and two men) who had not had to cope with the irregular demands of freelancing for over a year: “It was like this impossible Rubik’s cube that I just don’t know how we ever did that […] that caused me a lot of stress” (M4); “…the night before, I got myself in a real, not a state, but I was just like, what do I need? what time do I leave? […] All of those things that I wouldn’t have batted an eyelid about before suddenly are a big deal, and I didn’t sleep very well” (M8).

Most participants prepared for returning to work by obtaining and practicing their parts in advance, which they would not necessarily have done pre-pandemic: “I’ve just said to people ‘look, I need to have a look at it, because I’m not, you know, not on form, and if I don’t have a look at it I won’t be able to play it, so please can you send me the music’” (M5). The lives of professional performers are intrinsically stressful (Kegelaers et al., 2021), and comments such as “A lot of musicians are going to find the next parts of life psychologically tough, mentally tough, because we haven’t done it for such a long time” (M8) suggest that resilience training for music students (Matei and Ginsborg, 2021) and specialist psychological support for professionals (Gross and Musgrave, 2020) are both warranted.

*Working in a COVID-19 safe environment*: All the participants were double-vaccinated and accepted the need for testing, although this could cause anxiety: “[before playing in a West End show] you had to go into a room to be tested and depending on what the result was, you could go to work or not […] I was really nervous […] I was thinking ‘if I’m positive then I won’t be able to work, and there won’t be any money this week’” (M3). While several were anxious about the risk of infection when traveling to work on the tube, none felt it was risky to perform as masks and social distancing were mandatory, although disturbing: “[Wearing a mask] you lose your peripheral vision, that you don’t realise that you have been using until you haven’t got it […] it’s hot, it’s quite hard to just stay calm and breathe” (M5); “String players generally are like those penguins in Antarctica in the winter you know, it’s like how close can you get? […] all you hear really [distanced] is yourself, and so people, the tendency is to play less, but then when you play less, everyone else hears less, so it’s a self-defeating situation” (L12). One wind player demurred, however: “We’re used to playing at a distance in an orchestra, that’s what we do” (L8).

Most participants (*M* = 12, *L* = 7) reported that the length of concerts had been reduced to an hour with no interval. To mitigate smaller, socially distanced audiences, concerts were repeated but with no additional fee for the musicians: “Now we have to play the concert twice […] for the same amount of money, and that’s slightly insulting” (L11). Social distancing rules affected not only their working conditions but also its social aspects, shown in the first study to be very important to musicians: “…what’s really awful is there’s no social life. I mean you can’t go to the canteen and have a cup of tea and biscuit in the break […] you just sit there in dead silence in the studio […] it’s a horrible experience” (L8); “…that comradeship between you and your colleagues, a lot of it just hasn’t happened at all, it just hasn’t been possible […] I suppose it has felt quite lonely at times” (M6).

All but one of the participants had been involved in *online and live-streamed performances*, albeit generally unpaid (see *Maintaining identity as a musician*, above) and had developed home-recording skills. Some would not have done so, had it not been for the pandemic, and were glad: “…you know you’re taking care of the whole damn lot, the sound, the production […] I was quite proud that I managed to do that […] I mean I’m a complete virgin to all that kind of thing” (L6). For others, performing online was fraught with problems: “…just putting it together was the worst nightmare of my life, in fact we didn’t manage to put it together. In the end we had to send [the recording engineer] all the bits at five in the morning, and it was just the most stressful thing” (M2). Technological hiccups during live-streamed performances were experienced as particularly frustrating: “I had [the software] or whatever it’s called. I tested it, I got it to work, it was very painstaking, and then on the day, it just wouldn’t, Facebook wouldn’t link with [the software], and it just wouldn’t work” (M2), “[for a planned live-streamed independent orchestral concert] there was a problem with the, with the line-out, with the recording, so nobody saw it really. It was such a shame” (L3). In this case, no tickets were sold and consequently the players received no fee. Five mid-career participants described the difficulty of trying to make money out of online performing, an issue currently being addressed by the Fix Streaming campaign (Musicians’ Union, MU, 2022b).

### Maintaining Wellbeing/Coping Strategies

All the participants reported that the past year had been *emotionally challenging*; to maintain their wellbeing, they had used a variety of coping strategies including *support networks of family and friends*, *cognitive strategies*, *routines,* and *exercise and physical health*. Although, after a year, they were no longer reeling from the shock of losing their much-loved livelihoods, and their distress and anxiety were generally less intense, more than two-thirds of the participants in the present study (*M* = 11, *L* = 5, 10 women and six men) described some extremely *emotionally challenging* periods, reflecting the recent finding that women experienced greater psychological distress during the pandemic (Di Blasi et al., 2021): “Lockdown has been the equivalent of being locked in a padded cell, so you were really shut away with your own thinking and you know, I think if there was anything that was not right in your soul, you’d find, it would start, the worm would start to eat straight through you” (M12); “I reached a point where I was really, really struggling to function at all […] on those days I’ve sat in bed because I couldn’t do anything else” (M5). Several late-career participants feared that they would not be able to resume their careers: “You put up an armouring against it just to survive, just to be able to protect yourself from the pain of it all […] you get really tired of saying ‘*hopefully* we’ll be able to do it next year,’ ‘*hopefully* it will be alright,’ well we said that last year!” (L6). According to recent findings, women have been particularly vulnerable to loneliness throughout the pandemic (Bu et al., 2020), and this was reported by three women without partners or family: “I just sit there thinking ‘god I’m just on my own’… feeling flat and it’s really hard to pick yourself up” (M3). Again, specialist psychological support for these groups is warranted.

More than two-thirds of participants (*M* = 9, *L* = 7) valued *support networks of friends, family, and colleagues*. Four mid-career participants reported obtaining professional counseling during the pandemic. Although organizations such as Help Musicians, the MU, and the Incorporated Society of Musicians provided free online counseling sessions, only one participant reported using them; this suggests they could be advertised more effectively. Of the 10 participants with dependent children at home (*M* = 6, *L* = 4), all six mid-career participants (three men and three women) described the stress of looking after young children while simultaneously trying to find a way of earning a living: “It’s been challenging timewise in you know, finding people to look after him, that’s been hard when we’ve been both having to teach online” (M3). Commenting on the difficulty of dealing with home-schooling, another said: “…that home-schooling rubbish, it’s luckily so hard to remember […] whatever it was, was not the best” (M10); this illustrates the finding that the pandemic put intense strain particularly on those with younger families (Spinelli et al., 2020). The lockdowns did, however, offer an opportunity for families to spend more time together, and most of these participants (*M* = 5, *L* = 3, four men and four women) enjoyed seeing more of their children: “the real joy of having family around and having more family time […] so it’s a very heady cocktail of emotions” (L9). Others were coping with older parents’ ill-health at a time when there was little access to healthcare and, reflecting evidence of increased break-ups and divorces during the pandemic (BBC, 2020), three participants reported that their marriages or long-term relationships had collapsed: “I know for a fact that I’m not the only one going through a divorce, erm, I think it has put a strain on a lot of people and opened cracks that were already there” (M11).

The use of *cognitive strategies*, such as positive and soothing self-talk, was described by two-thirds of the participants (*M* = 9, *L* = 5, seven men and seven women): “I know it’s slow for everybody […] it’s not like everyone else is suddenly back to normal and I’m not” (M4). Others reframed their experiences (*M* = 5, *L* = 5): “You just have to keep saying that this isn’t forever, this is a very temporary thing […] It will be better next year” (M2). The remainder were not aware of using any strategies: “I just sort of fly by the seat of my pants and worry a lot” (M5). Again, specialist psychological support is warranted for this group.

Nearly half the participants (*M* = 6, *L* = 6, three men and nine women) found the use of *routines* very useful, particularly for practice motivation: “I’d [practise] every day, I’d do a technical routine, I’ve done it today and erm, that’s the only thing that keeps me going really. I just play it because that’s what you do” (L8). Some mid-career participants were surprised to find themselves enjoying a new weekly routine: “…suddenly having this real structure of you have a weekend, every week you will get the weekend, I’m just not used to that!” (M2). Listening to music was vital to three late-career participants: “I still connect with the music […] I just feel that love inside of me Susanna, that love of music inside me and that gets me through it” (L11). By contrast, a mid-career participant felt she had to distance herself from it: “It’s very hard to listen to music when you can’t play, when you haven’t got a hope of getting anywhere near because nothing’s happening […] it’s a bit depressing when you just feel that you might not do it again” (M2).

Two-thirds of participants (*M* = 7, *L* = 7, five men and nine women) had consciously engaged in regular physical *exercise*, such as yoga, Feldenkrais, walking, cycling, gardening, and lifting weights, to maintain their *physical fitness.* As in the general population (Khubchandani et al., 2022), a third of the participants reported having gained weight during the pandemic. Nevertheless six (*M* = 2, *L* = 4, three men and three women) had improved their eating and drinking habits because they were working less and spending more time at home: “I’m much fitter, I’m eating at home, I’m eating decent food, I’m not eating takeaways, I’m not drinking anything like as much beer as I usually do” (L8). Others were drinking more, however: “It’s like well ‘fuck it, I need a treat and I want a glass of wine, I’m going to have a glass of wine,’ stuff it, you know, I’ve got to do something nice for myself” (L6). Three (*M* = 2, *L* = 1) tried to limit their drinking: “I’m susceptible to depression anyway, so made a sort of an almost a rule for myself at the beginning of lockdown that I would, I wouldn’t just drink on my own at home” (M8). Pre-pandemic evidence that alcohol abuse is higher among musicians than in the general population (Crosby and McKenzie, 2021) suggests that it would be worth training music students to develop healthy lifestyles (Matei et al., 2018).

The use of social media might be considered a coping strategy. While a third of participants found it useful for publicizing projects, most felt ambivalent about it, and three mid-career participants described it as increasing their anxiety: “Actually a big factor that affected anxiety levels was social media last year with me, I had to come off it for a while” (M3). This reflects the findings of recent research (Gao et al., 2020) that social media can have both positive and negative influences on affect.

### Growth

Despite the challenges of the previous year, many participants reported experiences of *Growth.* These were grouped into three sub-themes: *New opportunities and skills, Resourcefulness,* and *New insights*; in addition, a further set of sub-themes associated with early signs of *Post-traumatic growth (PTG)* was identified.

More than two-thirds of participants (*M* = 10, *L* = 5, six men and nine women) had developed *new opportunities and skills* in the past year. Three mid-career participants had started businesses. One had become a distiller of fine gins and vodkas: “We’ve taken a lease on a little industrial unit […] we’ve now got our, our rectifiers and compounders’ licence […] it’s been quite a steep learning curve […] we’re sort of, we’re now distillers” (M7). The second was selling their landscape paintings, and the third had completed an online TEFL course, moved to a European country, and set up a new business combining English teaching with musical activities for young children. Among the other mid-career participants, one had started working as a writer and editor for a music magazine, one was a regular volunteer at a vaccination center, and one had learned to play the saxophone so that they could accept a doubling job in a West End show: “I ended up learning the tenor sax in lockdown in November just for this show […] in the past I would have said ‘no, I can’t do that’” (M3). Eight participants (*M* = 5, *L* = 3, three men and five women) described acquiring new online music production skills: “learning to use Logic, you know the recording software, that’s all been positive professional development that I wouldn’t have bothered to do otherwise” (M10).

The lockdowns had given four late-career participants in their mid-50s the opportunity to pursue music-related interests they had been considering for a number of years but previously not had a chance to develop. One had enrolled in a two-year course to retrain as a counselor in creative arts therapies, the second had started to focus on music making with children and adults with disabilities, the third had begun training to become a mindfulness teacher, and the fourth had worked as a museum curator and developed research into music and science. One late-career participant had also worked on developing their public speaking and presentation skills through attending toastmasters’ public speaking courses, and another participant in their mid-60s described composing music for the first time. While all the mid-career participants hoped that they would be able to combine their new non-performing interests with performing in future, some of the late-career participants said that their new skills might ultimately enable a transition out of professional performing: “I love music, but I actually even more than that, I love the creative process […] do I want to continue to try and make my whole living as a player, or not? […] it’s that connecting with other people deeply is really what makes me tick” (L4).

*Resourcefulness* is defined as the ability to find quick and clever ways to overcome difficulties (Oxford English Dictionary, 2022). It was evident in the interviews with most of the mid-career and some of the late-career participants (*M* = 11, *L* = 4, six men and nine women): “I’ve learned that when the chips are really down […] I am resourceful and so one career or one method of paying for everything, for life, just ceased, and I was resourceful enough to find another thing to make it work” (M8); “I did the Uber-Eats delivery because you know, it’s quite an ego-crushing thing to do to go and get a […] like a crap job essentially […] but I feel good that I did that because you know it’s a positive thing to do, to show that you can, you can adapt” (M10). While the older late-career participants in the first study had shown emotional as well as financial resilience, three of the four older late-career participants in the present study now seemed anxious, confused, and lacking direction, by comparison with the mid-career group: “I feel like I’ve done so little it’s […] like there’s so much I can do, could do” (L3).

Most participants (*M* = 10, *L* = 8, eight men and 10 women) described discovering *new insights and self-knowledge* during this period. Those of the mid-career participants focused mainly on their future careers and their desires to fulfill their aspirations as professional performers: “You know, my heart was full, I realized you know, even if I was being paid what was a pretty derisory sum, I felt ‘yeah, this is what I’m supposed to be doing, you know, I can do this really well, and I make a great contribution’” (M12). The late-career participants’ insights, by contrast, were centered more on teaching and helping others: “I’ve got experience and actually it’s really valuable to teach at this point you know” (L11). Such comments support lifespan models of musicians’ careers proposing a shifting focus, from the individual’s own performing achievements in mid-career to teaching and taking social responsibility later on in their careers (Manturzewska, 1990; López-Íñiguez and Bennett, 2020). Some of the older late-career participants were ready, in theory, to make way for younger players: “People like me really should be stepping back to make way for younger players now […] I’ve had the luck of playing for 40 years, of doing all sorts of fantastic, interesting work. I don’t think I should be grabbing work that I don’t actually totally need” (L2). Yet none were prepared to say they were no longer professional performers: “A lot of musicians I know wouldn’t even think of retiring, they wouldn’t dream of it, most of them who have gone into retirement, the work just petered out gradually for one reason or another, they aren’t employed much anymore. I’ve never wanted that to happen, I’d rather make the decision myself, from a professional point of view […] I just don’t feel ready to do that, but then I’m not sure I feel ready to play […] it’s sort of funny, it’s a kind of twist in the saga, isn’t it?” (L3). This illustrates the confusion musicians may feel when they face the dilemma of how to retire from performing life and the need for help and guidance in the decision-making process.

Several mid-career participants described the attainment of new self-knowledge: “I’ve learned a lot about myself. I’ve learned a lot about what makes me happy […] I’ve also learned who my friends are” (M1). They also had a new appreciation of the importance of achieving a better work-life balance: “I will be putting myself and my family higher up that order […] not to put work first all the time” (M10).

### Post-traumatic Growth (PTG)

Tedeschi and Calhoun (2004) define intensely negative and unexpected events that severely challenge the individual’s fundamental assumptions about the world as *traumatic.* They propose that the struggle with great adversity can, however, be transformative in producing post-traumatic growth (PTG) in one or more of five domains: (1) *appreciation of life*, (2) *openness to new possibilities*, (3) *personal strength*, (4) *relating to others,* and (5) *spiritual change*, and that growth in even one or two domains can be profoundly influential. In the first study, a third of the participants (*M* = 6, *L* = 2) had experienced the sudden loss of their much-loved careers as traumatic. Although the present study was carried out only a year later, there were already some indications of PTG. For example, 10 mid-career participants had a renewed *appreciation* for aspects of their professional and their personal lives: “I just thought, now I’m just going to go home to a really amazing family, you know, my wife still loves me (laughs), I’ve got a son who is actually pretty spectacular” (M12).

Many mid-career participants who had previously been very distressed at the devastation of the careers for which they had worked so long and so hard were now *open to new possibilities*: “English teaching was a route, an avenue, a way of getting here [abroad] and you know, having an income […] I removed every single thing that was sort of stable, and I changed everything” (M9); “I set up an art business which […] without COVID I don’t think, well I wouldn’t have had time to build a website, start selling art you know, doing commissions” (M11).

Several participants reported discovering *personal strength*: “I am definitely much more resilient than I thought I was. And that’s, that’s a quite nice thing to be aware of you know” (M8). Some felt they *related to others* differently: “Before the pandemic I was still in a relationship which I was wanting out of, couldn’t find a way out, and I was just going from pillar to post […] it’s definitely made me clearer about what’s right for me” (L7). Others felt they had undergone *spiritual change*: “I no longer feel like it’s a good thing to be just fine flying around being busy […] looking like you are sort of coping with life well, because life’s not like that” (L4).

It is important to note that, after only a year, it was still early to be looking for signs of PTG. However, from the generally lower levels of distress among participants in the present study, and the number of new career developments and opportunities being explored, and the new insights attained, it would appear that many participants may already have taken the first steps on their path towards PTG.

### Latent Themes

Three additional latent themes were also identified: *awareness and concern for others*, *being an intrinsically motivated self-learner,* and *feeling fortunate*.

As in the first study, *awareness and concern for others* was evident in all interviews, with participants expressing particular concern for musicians at earlier stages of their careers: “It’s the 20-somethings who’ve been so fucked over […] I think our generation should be looking out for them constantly […] and giving them the breaks and giving them the support” (L9). Some (*M* = 4, *L* = 1, three men and two women) experienced “survivor’s guilt” about musicians who might have had even less playing work: “I’m quite lucky that I’ve had bits of work, you know, you almost feel bad about it, you know, for the people that don’t” (M2). Participants’ sensitivity toward others might be a reflection of the acute listening and communicating skills that are essential qualities for freelance musicians to be able to carry out their everyday work of playing together (Myers and White, 2012).

A second latent theme identified was *Being a self-motivated learner.* This was evident in the motivation of both groups of participants to continue practicing, despite the absence of performing opportunities, and also in their motivation to develop new skills and interests in novel areas. Interestingly, the participant who had become a distiller during the pandemic described trying to improve the taste of a new gin in the same way as he practiced his instrument: “I thought alright, what can I do to do it better? […] maybe it’s that musicians’ mind […] I need to have one particular thing to aim for […] wanting to make it better, and wanting to sort of find a way” (M7). A recent study found that being an independent, intrinsically self-motivated self-regulated learner is a common characteristic of successful classical musicians (López-Íñiguez and McPherson, 2020), suggesting the need to encourage students to develop this trait during training (López-Íñiguez et al., 2022).

Given the distress experienced by participants in the first study, we were surprised to find that all 21 participants in the present study reported feeling “fortunate” or “lucky”: “I’ve, I’ve made [having to take a job in a café] work, but I’ve been incredibly, I count myself incredibly lucky” (M8). This might reflect participants’ gratitude for having been able to work for so long in their hitherto successful and highly satisfying careers in a competitive field (Gembris et al., 2018), or perhaps, the pandemic raised their awareness of and sensitivity towards the struggles of others who were worse off. It could even be that those who achieve careers as classical musicians are inherently more optimistic; all three possibilities merit further investigation. Feeling fortunate in the face of challenging circumstances is a form of “tragic optimism” (Wong, 2019), which emphasizes hope and, like dispositional gratitude, has been found to be a significant predictor of wellbeing during the COVID-19 pandemic (Mead et al., 2021).

### Thoughts for Music Colleges, Musicians’ Support Organizations, and Employers

At the end of each interview, participants were asked—in the light of the impact of the pandemic on their lives—how music colleges could equip music students more effectively for the future, and what contribution could be made by musicians’ support organizations and employers. All the participants emphasized the need for training in versatility and resourcefulness, for musicians to “have [their] fingers in a lot of different pies” (M11), and not to think that it would be enough simply to excel on their instrument(s). Music students should acquire decent instrumental teaching skills and not see teaching as a lower aspiration than performing (Bennett, 2009); after all, teaching had provided many of the participants with their only regular, reliable source of income during the pandemic. They should develop technological and home-recording studio skills and be able to carry out basic maintenance, especially on wind and brass instruments. In terms of financial management, they should be able to do their own accounts and have a savings plan, no matter how variable their earnings: “Obviously a lot of people have been hit by this, and possibly had no savings at all” (L3). Several participants suggested that music colleges needed to do far more to help musicians develop skills for psychological and physical wellbeing, which had been lacking even before the pandemic: “[music colleges] need trained coaches that can give the kind of guidance to musicians that professional sports people have. We don’t have that, and I think that’s a failing” (L6).

When asked about the contribution of employers, several participants mentioned their appreciation of being kept informed by companies they had previously worked for, even if they were currently unable to offer them any work. Others complained about lack of reciprocity (see *Cancelations*, above): musicians are expected to be flexible but not orchestral managements. Many felt that the MU was not doing enough to protect its members: “I wish the Musicians’ Union were somewhere that I could turn to for help, but I don’t feel that they would help me” (M1).

Pre-pandemic, more than half the participants (*M* = 8, *L* = 3) had used a diary service—an independent service through which orchestral fixers can book players for work. Unable to justify the expense when there was no work coming in, two participants had given up their diary services during the pandemic: “I’m really sorry I just can’t afford that any more […] I’d rather feed the kids” (M7). Other participants, however, praised diary service managers who had waived their fees, offered free help with CV-writing, and were available to chat on the phone: “They were and they are incredibly supportive […] it felt like we really were part of the same community […] I can’t speak highly enough of them, they’ve been absolutely fantastic” (M8). Petriglieri et al. (2019) describe self-employed workers as lacking a “holding environment” (p.132), a space in which work anxieties and tensions can be processed and employees can maintain their sense of identity. They suggest that the provision of holding environments could transform self-employed workers’ “precariousness into a tolerable and even generative predicament” (Petriglieri et al., 2019, p. 132). It appears that diary services served as holding environments during this period.

## Discussion

Our first study explored the impact of the pandemic on the lives of 12 established mid-career and 12 late-career (“seasoned”) freelance self-employed orchestral musicians in a series of interviews conducted in May and June 2020. The present study followed up all the mid-career and nine of the late-career participants in a second series of interviews conducted between June and August 2021. The main finding of the first study was that, although all the participants were anxious about their futures, having experienced the sudden loss of their much-loved and successful careers, the late-career group demonstrated emotional and financial resilience while the mid-career group was in crisis, anxious, and confused about their identity as musicians. The main finding of the present study is that, although freelance performing work was still scarce, the positions of the two groups had changed. All the mid-career participants remained committed to their performing careers. By contrast, four younger late-career participants, aged 54–59, had developed interests and acquired skills in aspects of music that didn’t necessarily involve performing and that might ultimately lead them to leave the profession as they had known it. Meanwhile the four older late-career participants, aged 66 and over, feared that the break from performing enforced by the pandemic meant that they might already, *de facto*, have retired. We now discuss the findings with reference to the precarity of working as a freelance orchestral musician (Bartleet et al., 2019; Willis et al., 2019; Caza, 2020), lifespan models of musicians’ careers (Manturzewska, 1990; Bennett and Hennekam, 2018; López-Íñiguez and Bennett, 2020), self-determination theory (Ryan and Deci, 2000), and the concepts of resilience (Bonanno et al., 2011), resourcefulness (Rosenbaum, 1983) and PTG (Tedeschi et al., 2018), and their implications.

Even before the pandemic, self-employed freelance orchestral musicians experienced precarity and insecurity (Teague and Smith, 2015; De Witte et al., 2016; Bartleet et al., 2019; Willis et al., 2019; Gross and Musgrave, 2020). The sudden loss of work at the beginning of the pandemic left many in an extremely vulnerable position both financially and emotionally, unable to earn a living and missing their careers (Cohen and Ginsborg, 2021; Spiro et al., 2021). Pre-pandemic, the authors of several studies had recommended music colleges to ensure that students enter the music profession with realistic expectations, emphasizing that salaried positions as a member of an orchestra are few and far between, and that it is very difficult to earn a living solely from performing; musicians should, rather, be prepared for varied portfolio careers, working as part of the gig economy (Bennett, 2009; Bartleet et al., 2019; Caza, 2020; López-Íñiguez and Bennett, 2020). Participants’ inability to support themselves and their families at the outset of the pandemic suggests that their portfolios of skills were not sufficiently varied, and points to the need for music colleges to train students not only for performing but also for teaching, recording, community music making, entrepreneurship, financial management, and running small businesses, so that the next generation of freelance musicians will be better able to cope with future challenges on a similar scale to those presented by the pandemic (Bennett and Bridgstock, 2015; López-Íñiguez and Bennett, 2020). In the past, music colleges have privileged the training of performers over teachers (Bennett, 2009), but teaching—the only reliable source of income for many of our participants—has a vital place in the freelance musician’s portfolio. As so many of our participants had little or no savings or pension provision, it would be worth musicians’ support organizations providing not only crisis referrals and help, as they have done throughout the pandemic, but also in-house financial management advice services, with details of flexible savings and pension schemes appropriate to the needs of a population whose earnings are often erratic.

Participants’ reports of last-minute cancelations of work without recompense, reduced fees, and lack of reciprocity with respect to musicians’ and managements’ flexibility suggest that freelance musicians need more effective representation to protect their working conditions. Discussions between representatives of both parties should take place as soon as possible to draw up an agreement on what constitutes reasonable and appropriate behavior, on both sides, in exceptional circumstances.

Although music listening and the use of music to maintain wellbeing all increased during the pandemic (Carlson et al., 2021; Granot et al., 2021; Hennessy et al., 2021), neither government nor the general public appear to understand what it means to earn a living working in the music industry. A third of the participants in our first study were ineligible for a SEISS grant (Musicians’ Union, MU, 2020), for example, and the public remains unwilling to pay for online music consumption. In addition to calling for the government to provide financial aid for musicians through the MU’s #InvestInMusicians, #FixStreaming, and #WorkingInTheEU campaigns, musicians’ organizations should also aim to educate the general public to have a greater appreciation and understanding of the expertise that is required to create the music they love but are reluctant to buy.

The continued commitment of our mid-career participants to pursue their performing careers supports lifespan models of musicians’ careers whereby musicians focus on their performing achievements in mid-career (Manturzewska, 1990; Bennett and Hennekam, 2018; López-Íñiguez and Bennett, 2020). Participants’ commitment to performing even though they could not play with others, live, in public, during the pandemic, illustrates the high level of classical musicians’ internal motivation shown in other studies (Appelgren et al., 2019; López-Íñiguez et al., 2022). Lifespan models also propose that musicians’ priorities change in late career, from their own achievements to passing on their skills and an increased sense of social responsibility. We saw this change in our late-career participants’ dedication to teaching and willingness to provide students with emotional support as well as extra lessons. Manturzewska (1990) describes a later stage in her lifespan model in which musicians “slowly but systematically retreat from professional activity. Musicians who are teaching or playing in orchestras retire” (p.137). She provides no information, however, as to how this process is or should be managed, suggesting that this is an area warranting further research.

In our first study, we examined the differences between the experiences of mid- and late-career participants through the lens of the PERMA model of wellbeing (Seligman, 2012). In the present study, we have chosen Self-Determination Theory (SDT) to explain the signs of growth we identified in our mid-career and younger late-career participants after a year, and the confusion we observed in the accounts of our older late-career participants. SDT posits “three basic innate psychological needs—competence, relatedness and autonomy—which when satisfied yield enhanced self-motivation and mental health and when thwarted lead to diminished motivation and wellbeing” (Ryan and Deci, 2000, p. 1). *Competence* is the need to gain mastery of tasks and to learn different skills; *relatedness* is the need to experience a sense of attachment and belonging to other people, and *autonomy* is the need to feel in control of one’s own behaviors and goals (Krause et al., 2019). Mid-career participants’ success in generating alternative sources of income and enjoying rare performing opportunities can be understood as satisfying the need for competence. They satisfied their need for relatedness by identifying themselves clearly as performing musicians, still part of the workforce despite the lack of performing work, and they satisfied their need for autonomy by forging ahead and exploring new opportunities despite the challenges posed by the pandemic. By contrast, the four older late-career participants were receiving state pensions and had no need to find alternative ways of earning money. Their need for competence was not satisfied, as two participants were injured and unable to perform and three were anxious as to whether they would be able to play again after taking such a long break. Their need for relatedness was not satisfied either, since most had not engaged very much in playing or teaching and were not sure if they still considered themselves performing musicians. Finally, they faced a dilemma: they had loved their careers as performers but were anxious about their competence; they didn’t feel they could retire or that they could return to performing. Accordingly, they tried to manage the situation by adopting a “wait and see” attitude but at the cost of not being in charge of their own goals or behaviors and thus failing to satisfy their need for autonomy. This finding underlines the need for older musicians to be provided with specialist careers advice and coaching to help them prepare for their departure from performing and plan the next stages of their lives.

In the first study, we found that our late-career participants demonstrated emotional and financial *resilience*, defined as the ability to maintain relatively stable, healthy levels of psychological and physical functioning when exposed to a potentially highly disruptive and adverse event (Bonanno et al., 2011). This finding confirmed those of other recent studies showing an association between older age and greater resilience at the start of the pandemic (Finstad et al., 2021). By contrast, we identified many examples of *resourcefulness* in the present study, especially among mid-career participants who appeared distressed and confused in the first study, as well as early signs of PTG. According to Tedeschi et al. (2018), “the struggle that leads to post-traumatic growth is not usually at first a struggle to grow or change, but rather to survive or cope. The growth tends to be unplanned and unexpected” (p. 5). We found evidence of PTG in both the intense anxiety and distress expressed by mid-career participants in the first study, and the novel, unexpected areas in which growth and new opportunities developed in the present study. Tedeschi et al. stress the difference between resilience and PTG in their conceptualization: resilience is the ability to bounce back and return to baseline functioning while PTG is transformative. There were few signs of transformative change in our older late-career players, however. Given recent reports of “the great resignation” (BBC, 2021b)—the phenomenon of retiring early in the light of insights attained during the pandemic (which itself may be an example of PTG)—the absence of transformative change among the older late-career participants in the study seems unlikely to be related to chronological age. While the resilience demonstrated by the older late-career participants in the first study appeared to be the product of their greater financial resources (they owned their own homes, had savings and regular income from state pensions), and their greater cumulative life experience which, according to the strength and vulnerability integration model (SAVI; Charles, 2010), enabled them to “navigate their worlds more successfully than younger adults” (Charles, 2010, p.1073), this initial financial and emotional resilience might have also been responsible for the absence of a sense of urgency in the need to create an alternative, meaningful work-life balance, which resulted in the feelings of confusion and lack of control expressed in the follow-up study. This supports existing evidence that failure to satisfy basic psychological needs in times of uncertainty such as the pandemic can produce confusion and distress (Vermote et al., 2022).

Maitlis (2020) argues that PTG can be facilitated by *sensemaking*, “a meaning-making process through which people work to understand unexpected or confusing events” (p. 405). Sensemaking takes place through the sharing of narratives with *attentive companions*, who have the “patience, empathy and the capacity to listen well” (Maitlis, 2020, p. 407). Given that we identified participants’ empathy and awareness of others in both our first study and the present study, and that the ability to build rapport and collegiality are essential parts of the freelance orchestral musician’s skillset, we recommend that musicians’ support organizations train suitable interested musicians to be “attentive others” and establish mentoring schemes in which they can facilitate PTG in those affected by crises such as the pandemic.

To create and sustain a successful career in music it is important to be a self-motivated learner (López-Íñiguez and Bennett, 2020); this also helped musicians maintain their motivation to practice during the pandemic (López-Íñiguez et al., 2022). Music colleges should therefore consider providing courses that help students to cultivate their capacity for self-motivated learning and self-efficacy—“the belief in one’s capabilities to organize and execute the courses of action required to produce given attainments” (Bandura, 1997, p. 3)—particularly as self-efficacy has been found to be positively associated with music students’ performance (González et al., 2018), more effective practice (Mornell et al., 2018) and health-promoting behaviors (Cohen and Panebianco, 2022). Feeling fortunate, the third latent theme, was a surprising finding, given the challenging circumstances in which the participants found themselves. Although existing examinations of the personality characteristics of classical orchestral musicians have consistently found that musicians exhibit higher levels of openness than non-musicians (Butkovic and Rancic, 2017; Vaag et al., 2017), there are no studies investigating trait optimism among freelance orchestral musicians, so far as we are aware, so this is an area meriting further research.

### Study Limitations and Conclusion

The first limitation of the present study is that it provided a snapshot of musicians’ experiences during a period when the number of COVID-19 cases was decreasing and a return to live performance appeared to be imminent. The situation changed, however, in the final months of 2021 with the emergence of the highly infectious Omicron variant resulting in more last-minute cancelations (The Strad, 2021). Although—at the time of writing—the government is behaving as though COVID-19 has become endemic, other variants may yet appear. If the disruption to participants’ lives and livelihoods in late 2021 and early 2022 is to be explored, a further follow-up study is required. The second limitation is that we didn’t investigate the experiences of early-career musicians, who were particularly badly affected by the pandemic (Spiro et al., 2021; UK Music, 2021), as we were more interested, initially, in its impact on musicians with already established, successful freelance careers. We now wish to interview a sample of these musicians. The third limitation is that the participants in both the first and the present study were self-selected musicians choosing to respond to invitations on social media to participate. This suggests that they are proactive and highly motivated, and as such, their experiences of the pandemic and its impact on their lives may not reflect those of other players.

In conclusion, the findings of the present study show that, a year on, the COVID-19 pandemic is continuing to have a huge impact on the lives of self-employed freelance orchestral musicians in the United Kingdom. This examination of the participants’ experiences over the longer term has served to shine a spotlight on the precarity of their working lives; on the need for music colleges to train future generations of musicians for more varied and robust portfolio careers; and on the need for greater support from the government and the general public. On the basis of our finding that the older late-career participants were no longer so emotionally resilient but, a year on, troubled and confused about their future, we recommend further research to inform the development of guidelines addressing both musicians’ and managements’ expectations as to when and how retirement should take place, and support for musicians in the later stages of their late career. We already knew that freelance orchestral musicians had to be determined, dedicated, organized, creative, and socially skilled team players. The latent themes we have identified from our participants’ accounts of developing new interests and the early signs of PTG that we have observed suggest that successful freelance orchestral musicians are also self-motivated learners, aware of others, and able to assume a positive, optimistic attitude in the face of challenges. Taken together, these attributes suggest that they possess an extremely powerful and transferable set of skills likely to be valuable in a huge array of working situations, whatever the future may bring.

## Data Availability Statement

The raw data supporting the conclusions of this article will be made available by the authors, without undue reservation.

## Ethics Statement

The studies involving human participants were reviewed and approved by the Research Ethics Committee, Royal Northern College of Music, Manchester, UK. The participants provided their written informed consent to participate in this study.

## Author Contributions

SC and JG designed the study and procedures and analyzed the data. SC carried out the interviews and drafted the manuscript with support from JG in all sections. Both authors contributed to the article and approved the submitted version.

## Funding

This research was carried out while SC was a Visiting Research Fellow at the Royal Northern College of Music, UK, and was supported by Help Musicians.

## Conflict of Interest

The authors declare that the research was conducted in the absence of any commercial or financial relationships that could be construed as a potential conflict of interest.

## Publisher’s Note

All claims expressed in this article are solely those of the authors and do not necessarily represent those of their affiliated organizations, or those of the publisher, the editors and the reviewers. Any product that may be evaluated in this article, or claim that may be made by its manufacturer, is not guaranteed or endorsed by the publisher.

## Footnotes

^1^Although restrictions were lifted in March 2022 and free testing was abandoned in April 2022, infection rates continued to rise; it is too early to say what the impact of these decisions will be on musicians and the general public.

^2^A payment to help with the living costs of people on a low income or out of work (https://www.gov.uk/universal-credit).

^3^ https://www.gov.uk/guidance/claim-a-grant-through-the-coronavirus-covid-19-self-employment-income-support-scheme

^4^A city in the south-west of England, far from London.

## Appendix A

### Interview Guidelines for Semi-Structured Interview (follow-up)

Could we start with an overview of how this past year has been for you, since we last spoke?

Prompts:

1. Are you working in music at the moment? How is that?
2. What do you have in your diary for the future? Are you (still) using a diary service?
3. Are you currently practising at all? If so, what? How is that?
4. What (if any) preparations are you doing for getting back to work?
5. How have you been managing financially?
6. When do you think you might be able to return to your pre-covid earnings from music?
7. How are you dealing with all the uncertainty?
8. What are your hopes/fears about continuing to work in the music profession?
9. How have your moods been?
10. What helps you to cope during this time?
11. Are there any positive things that have come out of this time for you?
12. What (if anything) have you learned/plan to take forward from this time?
13. In what way has Brexit influenced your professional life?
14. Are you vaccinated?
15. What do you think music colleges should be thinking when training young players?
16. What do you think employers should be thinking of?
17. Is there anything else you’d like to add or anything you think I’ve left out?

Thank you!
